# Identification of glioblastoma gene prognosis modules based on weighted gene co-expression network analysis

**DOI:** 10.1186/s12920-018-0407-1

**Published:** 2018-11-01

**Authors:** Pengfei Xu, Jian Yang, Junhui Liu, Xue Yang, Jianming Liao, Fanen Yuan, Yang Xu, Baohui Liu, Qianxue Chen

**Affiliations:** Department of Neurosurgery, Renmin Hospital of Wuhan University, 9 Zhangzhidong Road and 238 Jiefang Road, Wuchang, Wuhan, Hubei 430060 People’s Republic of China

**Keywords:** GBM, WGCNA, TCGA, Cox proportional hazards regression module

## Abstract

**Background:**

Glioblastoma multiforme, the most prevalent and aggressive brain tumour, has a poor prognosis. The molecular mechanisms underlying gliomagenesis remain poorly understood. Therefore, molecular research, including various markers, is necessary to understand the occurrence and development of glioma.

**Method:**

Weighted gene co-expression network analysis (WGCNA) was performed to construct a gene co-expression network in TCGA glioblastoma samples. Gene ontology (GO) and pathway-enrichment analysis were used to identify significance of gene modules. Cox proportional hazards regression model was used to predict outcome of glioblastoma patients.

**Results:**

We performed weighted gene co-expression network analysis (WGCNA) and identified a gene module (yellow module) related to the survival time of TCGA glioblastoma samples. Then, 228 hub genes were calculated based on gene significance (GS) and module significance (MS). Four genes (OSMR + SOX21 + MED10 + PTPRN) were selected to construct a Cox proportional hazards regression model with high accuracy (AUC = 0.905). The prognostic value of the Cox proportional hazards regression model was also confirmed in GSE16011 dataset (GBM: *n* = 156).

**Conclusion:**

We developed a promising mRNA signature for estimating overall survival in glioblastoma patients.

**Electronic supplementary material:**

The online version of this article (10.1186/s12920-018-0407-1) contains supplementary material, which is available to authorized users.

## Background

Glioblastoma multiforme (GBM), the most prevalent and aggressive primary intracranial tumour, displays heterogeneity, rapid proliferation and extensive invasion, with a median survival of approximately 15 months [1, 2]. Therefore, developing appropriate and effective biomarkers to predict prognosis is crucial for glioblastoma patients. Previous genomic analyses of glioblastoma have identified some molecular markers, including epidermal growth factor receptor (EGFR), platelet-derived growth factor receptor alpha (PDGFRA), vascular endothelial growth factor (VEGF), insulin-like growth factor 1 (IGF-1), P53 and isocitrate dehydrogenase 1 (IDH1), and X-linked alpha thalassemia mental retardation syndrome gene (ATRX) [3, 4]. In addition, methylation levels of the promoter of O6­methylguanine­DNA methyltransferase (MGMT) are related to sensitivity of temozolomide therapy and the prognosis of patients. The 1p/19q loss is another prognosis marker and indicates a better prognosis [5].

With the development of high-throughput sequencing and bioinformatics, abundant sequencing data provide a remarkable opportunity to detect glioblastoma-associated key genes, networks and pathways. However, identification of these features remains challenging. Weighted gene co-expression network analysis (WGCNA) is a system biology method used for describing the correlation patterns among genes and finding highly correlated modules. In this study, we performed WGCNA for RNASeq data derived from The Cancer Genome Atlas (TCGA) and reconstructed gene co-expression networks. Then, we identified gene modules related to survival and recurrence time. Using Kaplan-Meier survival analysis and multivariate Cox regression analysis, we identified an independent prognostic model. This finding provides new insights into the molecular mechanism of GBM.

## Methods

### Data download and pre-processing

RNA sequencing data (RNA-Seq2 level 3 data) from human glioblastoma samples were obtained from the TCGA data portal (https://portal.gdc.cancer.gov), which contains 152 glioblastoma tissues [3]. These data were updated to January 28, 2016. According to the instructions for WGCNA, fragments per kilobase per million (FPKM) is recommended as the data type for subsequent analysis. As some genes without significant changes in expression between samples will be highly correlated in WGCNA, the 5000 most differentially expressed genes were used in the following WGCNA studies. The clinical metadata of 152 samples were also downloaded and filtered for useful information. Because the clinical data of TCGA database was constantly updated, the survival time of the death patients was more accurate. The age, gender, survival time and recurrence time data of 75 deceased patients were selected and divided into two groups according to the median (Table 1). Subtype data of 152 samples was downloaded from GlioVis database (http: //gliovis.bioinfo.cnio.es/) [6]. The GSE36245, GSE16011 and GSE50161 datasets were included in the study, and both originated from an Affymetrix Human Genome U133 Plus 2.0 Array [7–9]. GSE36245 dataset only contained 46 glioblastoma samples, so it was used to validate whether the modules which are obtained from TCGA database were reproducible. GSE16011 dataset (GBM: *n* = 159) was used to validate whether the Cox proportional hazards regression model was reproducible. GSE50161 dataset (GBM: *n* = 34; Normal control: *n* = 13) contained glioblastoma and normal brain samples and was used to perform difference analysis.

**Table 1:** Information for 75 glioblastoma patients

TCGA Datasets	
Variables	Case number(*N* = 75)
Age	
< 59	38
> =59	37
Gender	
Female	55
Male	20
Survival time	
< 448	39
> =448	36
Recurrence time	
< 178	38
> =178	37

### Weighted gene co-expression network analysis and module preservation

**Fig. 1:**
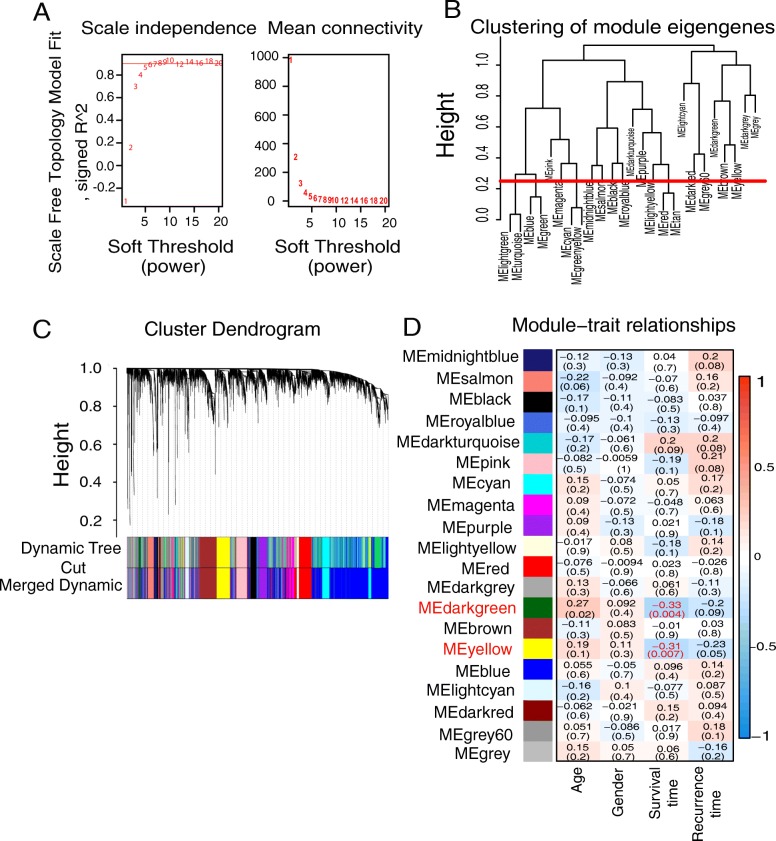
Weighted gene co-expression network of glioblastoma. **a** Identification of the soft threshold according to the standard of the scale-free network. **b** Dendrogram of consensus module eigengenes. The red line represented merging threshold. Modules with a correlation coefficient greater than 0.75 were merged. **c** Identification of a co-expression module in glioblastoma. The branches of the cluster dendrogram correspond to the 19 different gene modules. Each piece of the leaves on the cluster dendrogram corresponds to a gene. **d** Correlation between the gene module and clinical traits. The clinical traits include age, gender, survival time and recurrence time. The correlation coefficient in each cell represented the correlation between the gene module and the clinical traits, which decreased in size from red to blue. The corresponding *P* value is also annotated

WGCNA was performed using the R package WGCNA [10]. First, RNASeq data were filtered to reduce outliers. A co-expression similarity matrix was composed of the absolute value of the correlation between the expression levels of transcripts. For an unsigned network, the correlation coefficient between gene i and j was defined as Sij: Sij = |cor(i,j)|. The co-expression similarity matrix was then transformed into the adjacency matrix by choosing 7 as a soft threshold (Fig. 1a). A topological matrix was created using the topological overlap measure (TOM) [11, 12]. Finally, we chose the dynamic hybrid cut method, a bottom-up algorithm, to identify co-expression gene modules [13]. To identify the significance of each module, we calculated gene significance (GS) to measure the correlation between genes and sample traits. Module significance (MS) was defined as the average GS within modules and was calculated to measure the correlation between modules and sample traits (age, gender, survival time and recurrence time) [14, 15]. Statistical significance was determined using the correlation *P* value. The module Preservation function in the WGCNA R package was used to calculate the Z _summary_ to evaluate whether a module was conserved [16].

### Hub gene identification and module visualisation

Hub genes were identified using “network screening” within the R package WGCNA [10]. This method identifies genes that have high GS and MS. We selected the q. weighted < 0.01 as a cutoff to obtain the hub genes [17]. The targeted module visualisation was performed using Cytoscape3.5.1. Cytoscape is an open source software for visualising molecular interaction network (http://www.cytoscape.org/index.html) [18].

### Functional enrichment analysis

Gene ontology (GO) and pathway-enrichment analysis (Kyoto Encyclopedia of Genes and Genomes (KEGG)) were performed using the R package clusterProfiler (https://guangchuangyu.github.io/clusterProfiler) [19–21]. Enriched ontological terms and pathways with *P* < 0.05 were selected.

### Cox proportional hazards regression model

The prognostic value of each hub gene was first assessed by univariate Cox proportional hazards regression. Then, statistically significant genes were used to construct the multivariate Cox regression model as follows: Risk score = (0.2844* expression level of oncostatin M receptor (OSMR)) + (− 0.1682* expression level of SRY-Box 21 (SOX21)) + (1.3462* expression level of mediator complex subunit 10 (MED10)) + (0.3776* expression level of protein tyrosine phosphatase, receptor type N (PTPRN)). Glioblastoma samples were divided into high-score and low-score groups based on the median of the risk score. K-M survival curves were generated to assess the prognostic value of the model using the R package “survival” (https://CRAN.R-project.org/package=survival). The receiver operating characteristic curve (ROC) was generated to assess the accuracy of the model with the R package “survivalROC” (https: //CRAN.R-project.org/package = survivalROC) [22].

### Statistical analysis

The Pairwise t tests and Tukey’s Honest Significant Difference test were used to perform differentail analysis. All statistical tests and graphing were performed using RStudio (www.rstudio.com) and GraphPad Prism 7.0. *P* values < 0.05 were considered statistically significant [23]. Statistical significance was indicated in the figures as follows: *P < 0.05, **P < 0.01 and ***P < 0.001.

## Results

### Pre-processing of TCGA RNA sequencing and clinical data

Glioblastoma RNASeq data were downloaded from TCGA and constructed into a matrix RNASeq with gene symbols as the rows and patient barcodes as the column names. Furthermore, expression estimates in less than 20% of cases were removed, and the top 5000 most differentially expressed genes were used in WGCNA studies. Simultaneously, the corresponding clinical data were also downloaded to relate co-expression modules to clinical phenotypes. After outliers were removed, we selected data from 75 deceased patients among the 152 samples, including 5000 genes (Table 1).

### Gene co-expression network analysis

WGCNA was performed to construct a gene co-expression network to identify biologically meaningful gene modules and better understand the molecular mechanism of glioblastoma. WGCNA defined gene modules as a set of genes with topological overlaps. The specific approach was to establish a hierarchical clustering tree based on dynamic hybrid cut. Each piece of the leaves on this tree corresponded to a gene, and the different gene modules were the branches of the tree. Identification of co-expression modules could facilitate identification of hub genes that drive and maintain important functions. Ultimately, 19 gene modules were identified. The grey module includes genes that were not assigned to any gene modules (Fig. 1b, c).

### Calculation of module-trait correlations in GBMs

To analyse the relationship between gene modules and sample clinical information, we used the module eigengene (ME) as the overall gene expression level of corresponding modules and calculated correlations with clinical phenotypes, such as age, gender, survival time and recurrence time. The yellow and dark green modules were significantly associated with survival time (Fig. 1d and Additional file 1: Table S6).

### Module preservation statistics

**Fig. 2:**
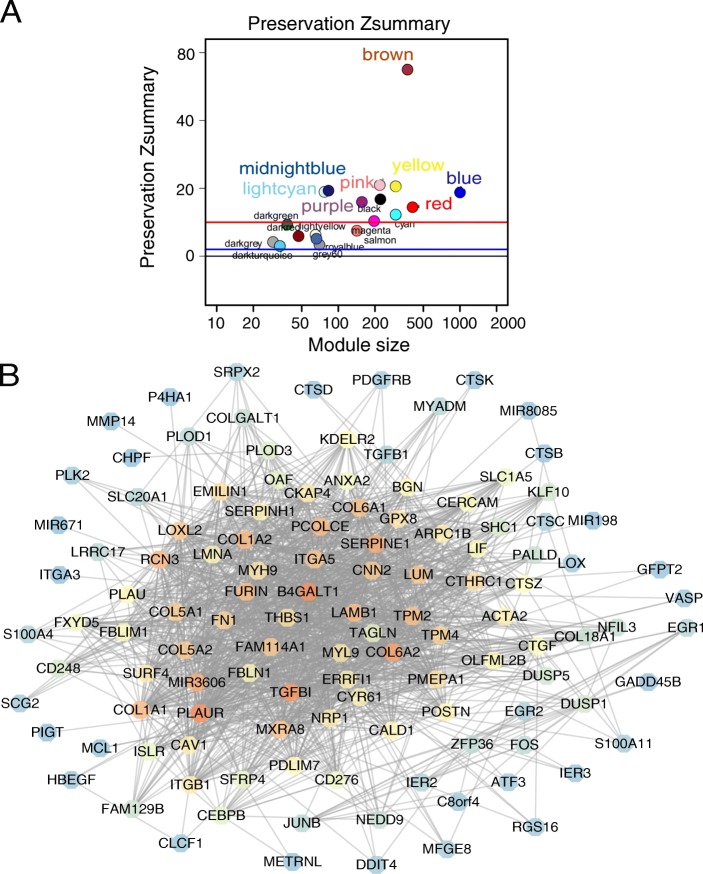
Module preservation and visualisation. **a** Module preservation statistics of TCGA modules in GEO modules (y - axis) vs module size (x - axis). **b** Visualisation of the gene co-expression network of the yellow module was generated using Cytoscape. Based on weight, not all genes corresponding to each module were represented

To validate whether the modules were reproducible (or preserved), we selected 4644 genes which from GSE36245 (GBM: *n* = 46) to construct a weighted gene co-expression network. Then, the Z _summary_ score was calculated to determine module preservation. Modules with a Z _summary_ score > 10 were regarded as preserved [24]. That is, the modules of the TCGA dataset also existed in the network of the Gene Expression Omnibus (GEO) dataset. The 10 modules were highly conserved, including the yellow module, while the dark green module was poorly conserved (Fig. 2a). Thus, we focused on analysis of the yellow module in the follow-up study.

### Identification of the hub gene

The function of the WGCNA R package, called network Screening, was used to search for the hub gene in the yellow module. We used the q. weighted < 0.01 and obtained 228 survival-related genes. These intramodular hub genes were centrally located in their respective modules and may thus be critical components within the modules [25].

### Module visualisation of network connections

To further depict the expression network of module genes related to survival time, we exported the co-expression network of the yellow module into Cytoscape. The nodes were defined as individual genes in the network, and edges were defined as the interactions between genes. As shown in Figures, the yellow module included 311 nodes and 21,557 edges. The hub genes of the modules were marked as orange nodes (Fig. 2b).

### GO and pathway-enrichment analysis of hub genes

**Fig. 3:**
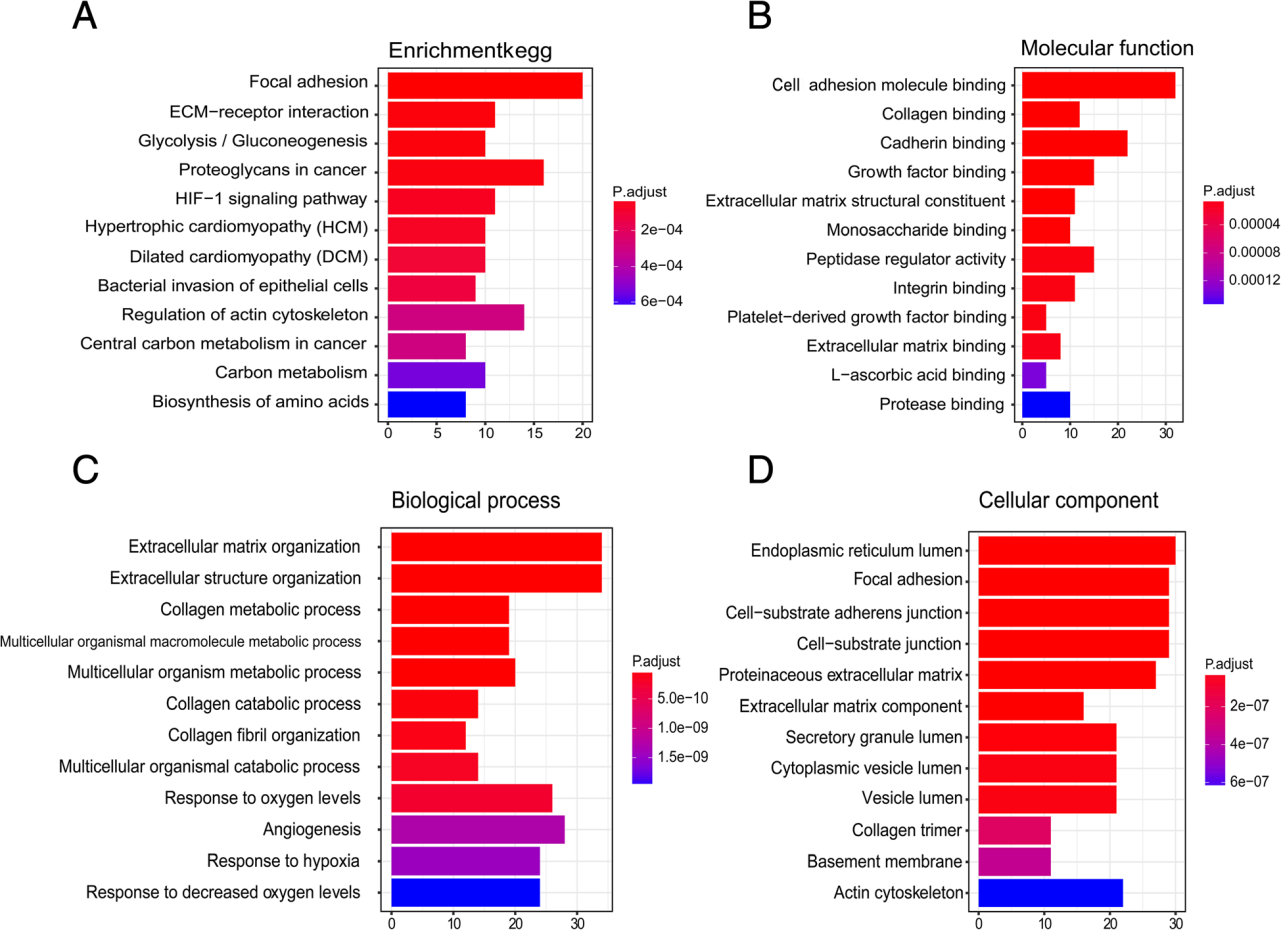
Enrichment analysis of hub genes. **a** Enrichment of Kyoto Encyclopedia of Genes and Genomes (KEGG) pathway analysis for 228 hub genes related to survival time. Molecular Function **b**, Biological Process **c** and Cellular Component **d** of Gene Ontology (GO) enrichment analysis were shown separately

To explore the cellular component (CC), molecular function (MF) and biological process (BP), we performed GO enrichment analysis. A total of 228 hub genes were significantly enriched in the following subclasses of GO classification (Fig. 3): focal junction (GO: 0005925, *P* = 3.17E - 15), cell adhesion molecule binding (GO: 0050839, *P* = 1.07E - 15), collagen binding (GO: 0005518, *P* = 1.56E - 11), extracellular matrix organisation (GO: 0030198, *P* = 2.67E - 20), and extracellular structure organisation (GO: 0043062, *P* = 1.10E - 21). KEGG pathway analysis showed that the top enriched terms were focal adhesion (hsa04510, *P* = 1.53E - 10) and ECM-receptor interaction (hsa04512, *P* = 1.39E - 07) based on *P* value. These results suggest that these genes were closely related to the cell adhesion function (Fig. 3a–d and Additional files 2, 3, 4, 5: Table S2–5).

### Construction of the cox proportional hazards regression model based on hub genes and Kaplan-Meier analysis

**Fig. 4:**
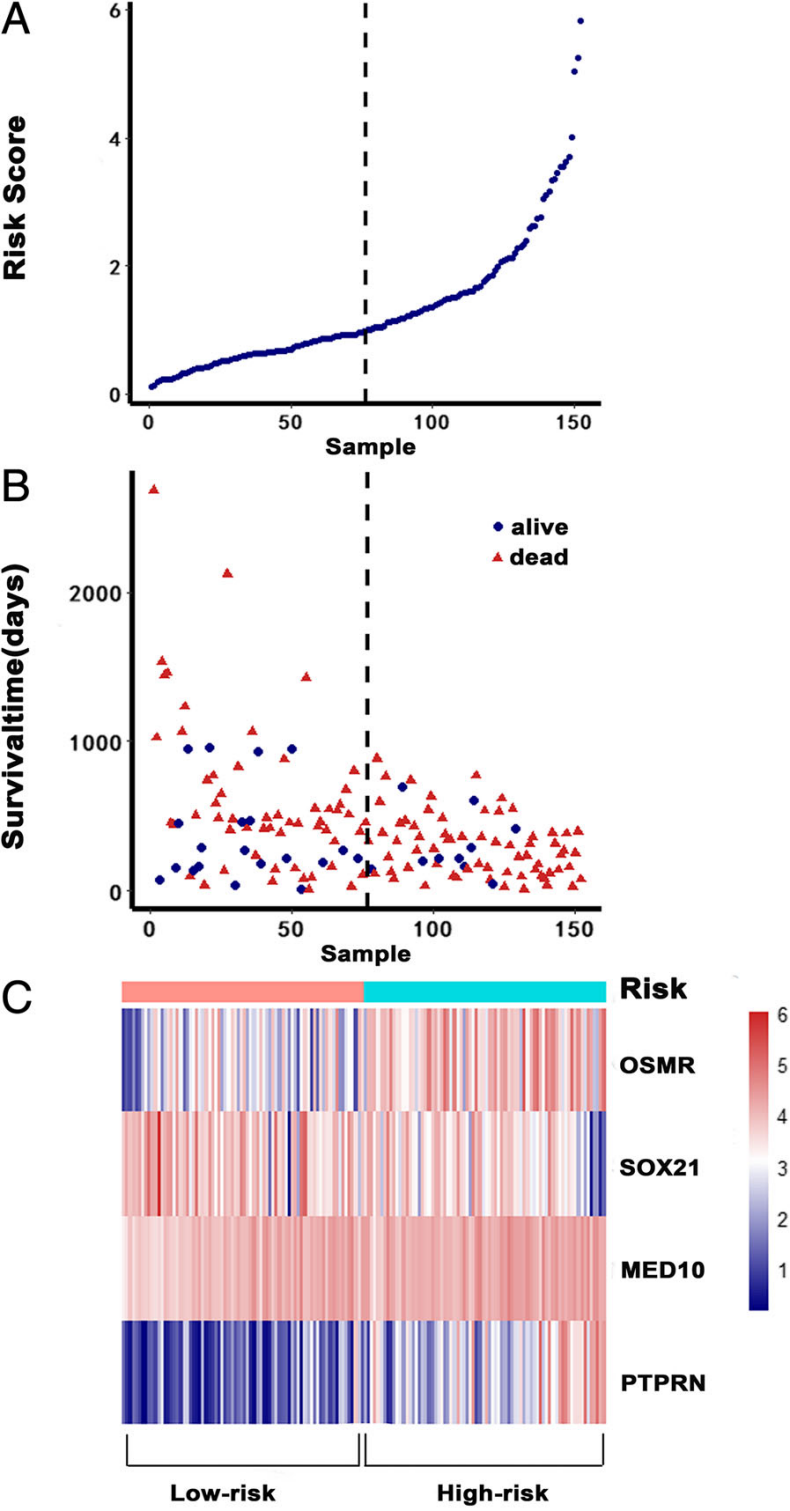
Cox proportional hazards regression model. **a** The risk score of the low-risk and high-risk groups. **b** Survival status and time of 152 glioblastomas. **c** Heatmap of the model genes

**Fig. 5:**
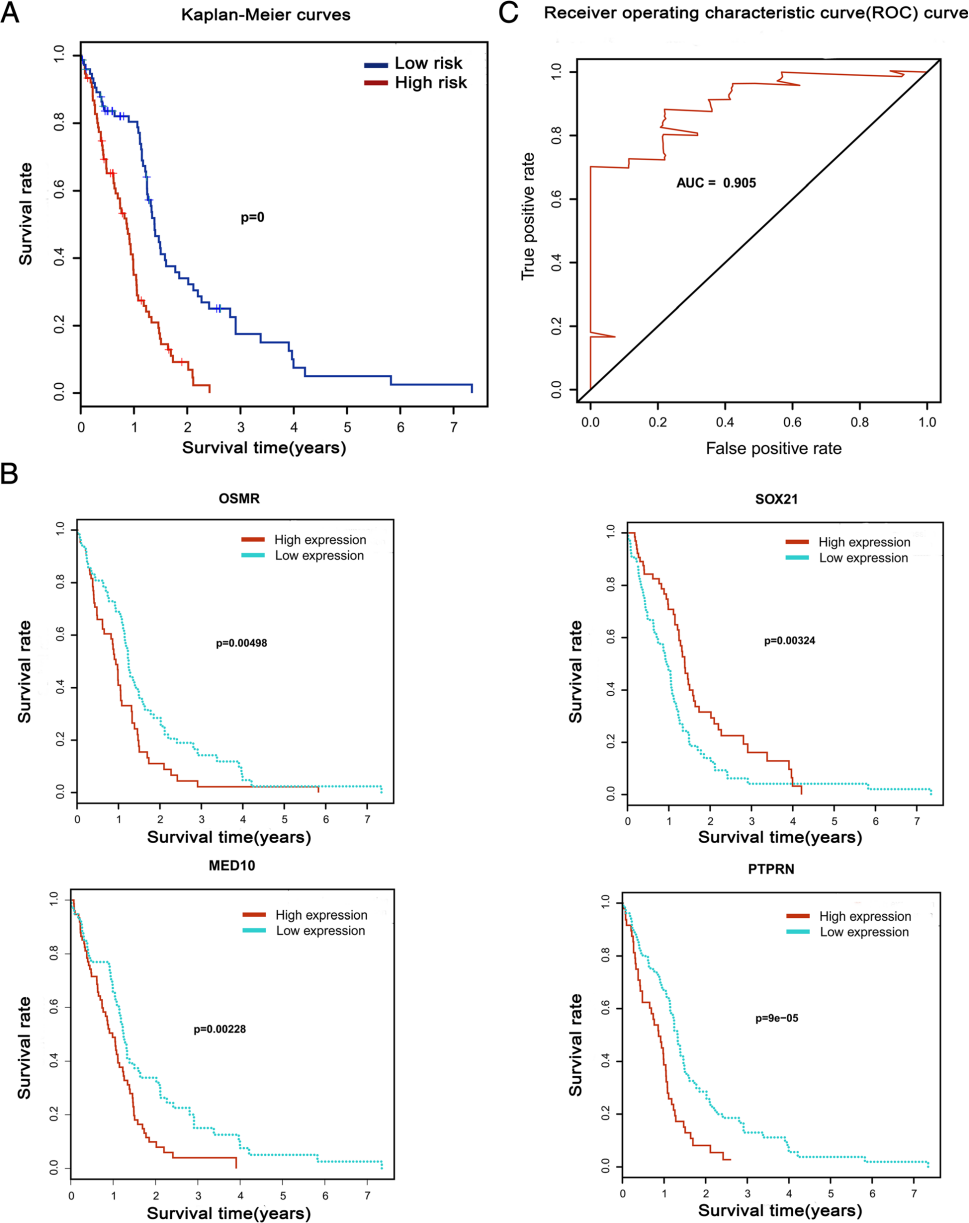
Kaplan-Meier curves and receiver operating characteristic (ROC). **a** Kaplan-Meier curves show that the high-risk group had greater mortality than does the low-risk group (*P* = 0). **b** Kaplan-Meier curves of the four different genes. **c** Time-dependent ROC curves indicated higher predictive value. The area under the ROC curve (AUC) was 0.905

We further narrowed down and selected the top 20 genes significantly related to survival time by univariate Cox analysis of 228 hub genes (Additional file 6: Table S1). Then, we used the 20 genes to perform multivariate Cox analysis and construct a Cox proportional hazards regression model from 152 glioblastoma patients. The risk score for predicting survival time was calculated with a formula based on the above mentioned four genes: risk score = (0.2844 * expression level of OSMR) + (− 0.1682 * expression level of SOX21) + (1.3462 * expression level of MED10) + (0.3776 * expression level of PTPRN) (Fig. 4a–c). We divided 152 patients into high-risk (*N* = 76) and low-risk (*N* = 76) groups according to the median of the risk score. The five-year survival rate of the high-risk group was significantly poorer than that of the low-risk group (Fig. 5a). The model was reproducible in GSE16011 dataset (Additional file 7: Figure S1 and Additional file 8: Table S7). Elevated expression of OSMR, MED10 and PTPRN was associated with an increased risk score, but SOX21 produced the opposite effect. The area under the ROC curve was 0.905 (Fig. 5c), indicating a higher predictive value. Moreover, K-M curves confirmed that the four genes could function as an independent predictive indicator for the survival of glioblastoma patients (Fig. 5b).

### Difference analysis of the four genes

**Fig. 6:**
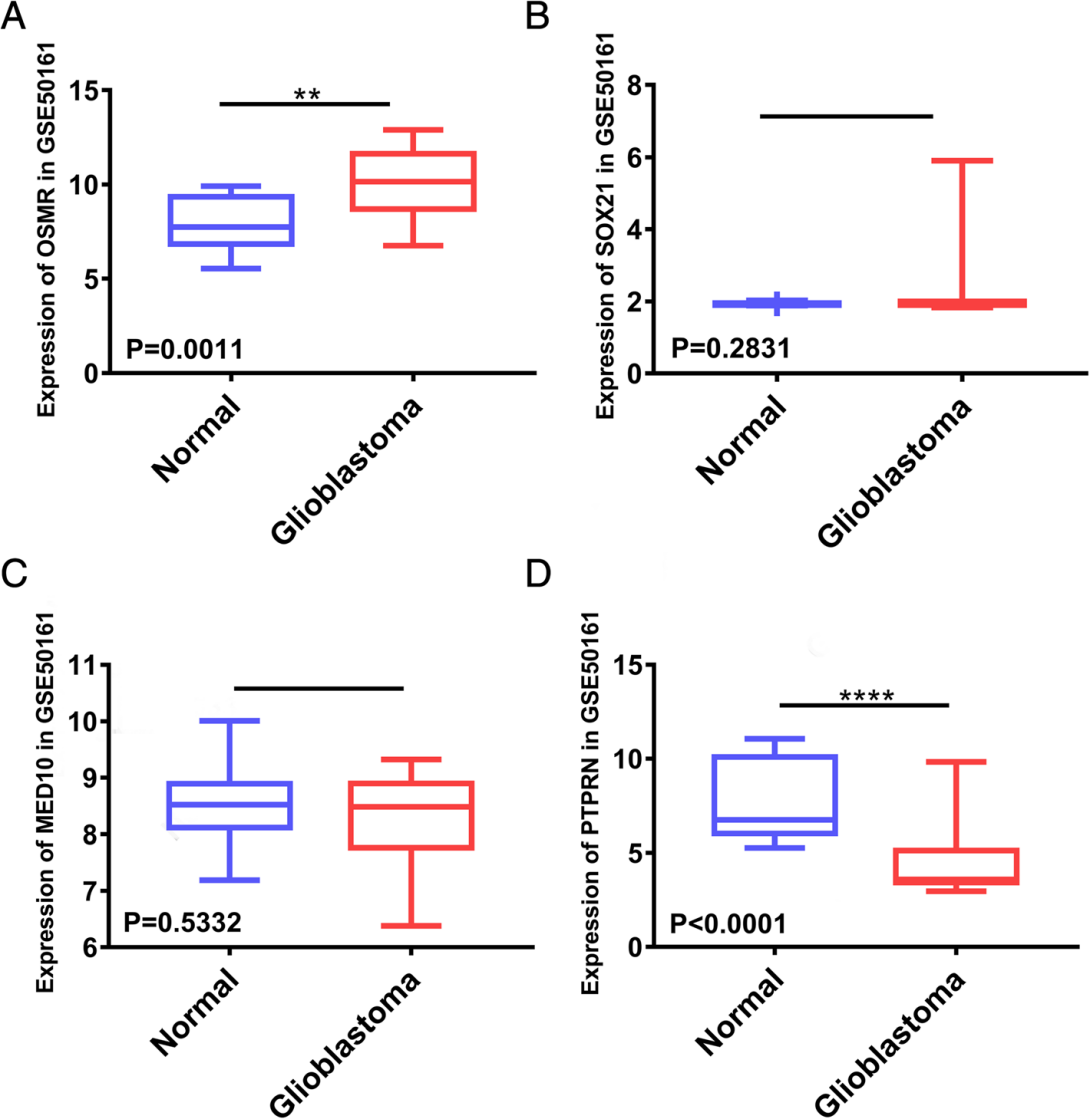
Expression of the four genes between normal and glioblastoma tissue in GSE50161. **a** OSMR; **b** SOX21; **c** MED10; **d** PTPRN; **P < 0.01, ****P < 0.0001

**Fig. 7:**
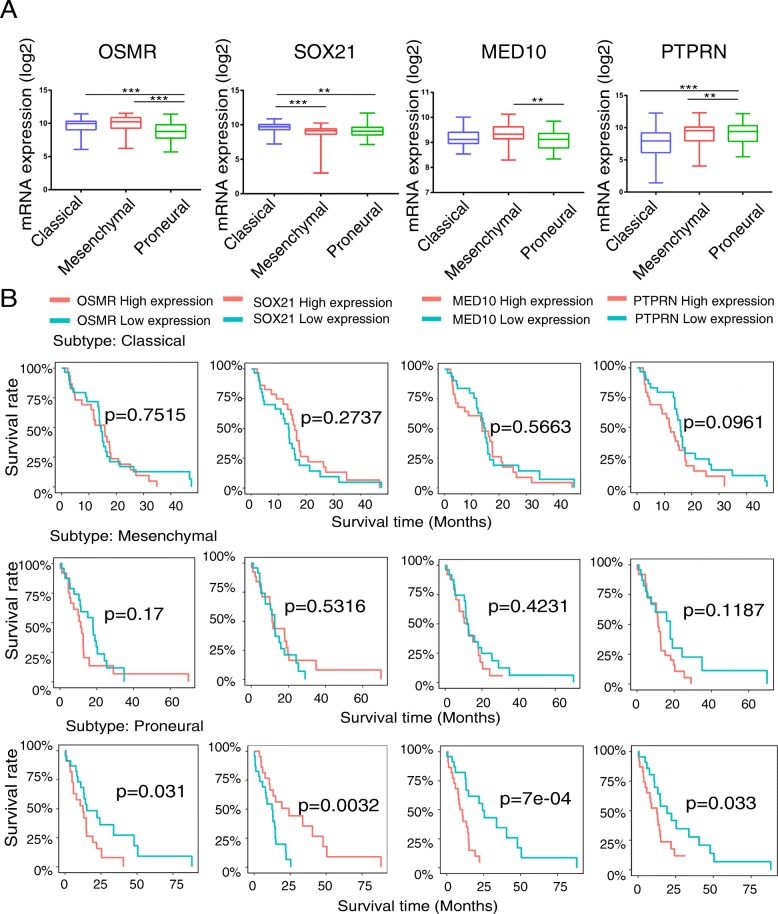
Expression of the four genes in different molecular subtypes of glioblastoma. **a** mRNA expression levels of OSMR, SOX2, MED10 and PTPRN in three molecular subtypes of glioblastoma. Statistical significance was indicated in the figures, **P < 0.01, ***P < 0.001. **b** Survival curves for the four genes set in different subtypes

To assess the expression level of the four genes between normal and glioblastoma tissues, we chose the GSE50161 datasets (normal brain tissue = 13, glioblastoma tissue = 34) to perform difference analysis. Interestingly, OSMR (*P* = 0.0011) and PTPRN (*P* < 0.0001) were differentially expressed (Fig. 6a, d), while MED10 (*P* = 0.5332) and SOX21 (*P* = 0.2831) were not (Fig. 6b, c). Subsequently, we assessed the mRNA expression levels of the four genes within each subtype (Classical, Mesenchymal and Proneural) [26]. The results showed that mRNA expression levels of the four genes in proneural subtype were significantly different from the other two subtypes (Fig. 7a). Meanwhile, the four genes had a better prognosis in proneural subtype (Fig. 7b).

## Discussion

Due to the diffusely infiltrative nature of glioblastoma, completely removing tumours is difficult, and these tumours also resist radiation therapy and chemotherapy. Thus, molecular studies, including various markers, are necessary to understand gliomagenesis and development. In addition, some molecular markers are important for determining molecular subtypes, identifying individualised treatments and judging clinical prognosis. For instance, overexpression or amplification of EGFR, mutations in IDH1 and IDH2 and phosphatase and tensin homologue (PTEN) mutations contribute to the pathogenesis of glioblastoma [27]. With the rapid development of high-throughput sequencing and bioinformatics methods, exploiting the great potential of RNASeq data requires new analytic approaches that move beyond gene difference analysis.

Instead of relating thousands of genes to a clinical trait, we used a recently developed methodology to construct a weighted gene co-expression network in 75 glioblastoma samples from TCGA, revealing survival time-specific modules (yellow, *p* < 0.01). As the most important gene in the module, the hub gene is the main feature of the gene module and closely related to the corresponding clinical information. Thus, we identified 228 intramodular hub genes based on GS and MS. The enrichment analysis of GO and KEGG showed that adhesion function and adhesion molecules accounted for the highest proportion of hub genes. These results can partly explain why glioblastoma tumours exhibit high invasiveness, and adhesion molecules can play an important role in gliomagenesis. By constructing the Cox proportional hazards regression model, we selected an optimal four-gene model (OSMR + SOX21 + MED10 + PTPRN) for prognosis prediction. Among the genes in this model, OSMR and SOX21 have been previously reported in glioblastoma studies [28–31]. OSMR encodes a member of the type I cytokine receptor family. OSMR forms a complex with EGFRvIII, the most common EGFR mutation that occurs in glioblastoma, and regulates glioblastoma tumour growth. Overexpression of OSMR and low methylation level was reported to have a poor survival time in GBM [28]. According to our research and previous reports [29], expression level of OSMR was higher in mesenchymal and classical subtypes than proneural subtype. SOX21, the counteracting partner of SOX2, functions as a tumour suppressor during gliomagenesis by negatively regulating SOX2 [30, 31]. Currently, MED10 is known only as a component of the coactivator for DNA-binding factors that activate transcription via RNA polymerase II. The protein encoded by PTPRN is a member of the protein tyrosine phosphatase family and may be involved in cancer initiation and progression [32]. However, MED10 and PTPRN have not been previously reported in glioblastoma-related studies. Each gene was confirmed to have independent prognostic significance. The difference analyses were performed in the GSE50161 datasets. Although MED10 (*P* = 0.5332) and SOX21 (*P* = 0.2831) exhibited no differential expression in glioblastoma and normal tissues, they may exhibit differential expression between glioblastoma and low-grade glioma. Thus, further studies are needed.

WGCNA used a statistical method to make the gene network consistent with the scale-free distribution; the resulting gene modules are more in line with biological phenomena and can be more finely divided. To date, there are a few similar studies on glioblastoma. Aoki K used the Cox proportional hazards regression model to investigate the effects of genetic alterations in 308 diffuse lower-grade gliomas (LGGs) and verified the results using the dataset from TCGA. The authors reported that IDH mutation, 1p19q deletion, Notch homologue 1 (NOTCH1) mutations and phosphoinositide-3-kinase regulatory subunit 1 (PIK3R1) mutations were significantly associated with poor prognosis in LGGs [33]. However, glioblastomas were not examined. Horvath S adopted WGCNA to detect oncogenic modules and confirm abnormal spindle-like microcephaly-associated protein (ASPM) as a potential molecular target in glioblastoma [34]. Yu X used protein expression data of development process of macaque rhesus brain and RNA-seq data of GBM to identify several prognostic genes [35]. Similarly, Xiang Y applied WGCNA and K-means algorithm in gene expression data of GBM obtained from the TCGA database and found some prognosis sub-networks [36]. But, compared to the K-mean clustering method, WGCNA can construct a gene co-expression network to identify the hub genes associated with trait-related modules directly. Whether the two methods are used simultaneously was reasonable needed to further research. In addition, similar studies using WGCNA to predict prognostic molecules are rare. These results indicate that further analysis of this module may provide more clues to understand the occurrence and development of glioma. However, this study has some limitations. First, we did not validate the prognostic value of the four-gene model due to the lack of survival data in the GEO datasets. Thus, prediction of prognosis using the four-gene model needs further verification. Second, we selected only 5000 genes for analysis in WGCNA. These transcript changes can not represent all the genetic changes in glioblastomas. By increasing the number of genes in the study, we can find more molecular targets and key pathways. Third, these results were only detected using bioinformatics analysis and needed further experimental verification. Overall, our study provide a new perspective to identify the potential molecules and therapeutic targets for glioblastoma.

## Conclusions

In conclusion, in this study we performed a WGCNA approach with GBM RNA-seq data from TCGA database to reveal a survival time-specific module. We also constructed a Cox proportional hazards regression model and identified four independent prognostic factors (OSMR, SOX21, MED10 and PTPRN). Although the specific mechanism remained to be studied, these genes could be considered as risk factors for GBM patients and novel therapeutics.

## Additional files

Additional file 1: **Table S6.** Specific data in Fig. 1d. (XLSX 10 kb)

Additional file 2: **Table S2.** Biological process of hub genes. (CSV 60 kb)

Additional file 3: **Table S3.** Cellular component of hub genes. (CSV 8 kb)

Additional file 4: **Table S4.** Molecular function of hub genes. (CSV 5 kb)

Additional file 5: **Table S5.** KEGG-enrichment of hub genes. (CSV 2 kb)

Additional file 6: **Table S1.** Top ten prognostic genes identified from Cox regression analysis. (DOCX 21 kb)

Additional file 7: **Figure S1.** Kaplan-Meier curves and receiver operating characteristic (ROC) in GSE16011 dataset. (TIF 2529 kb)

Additional file 8: **Table S7.** Follow-up information of GSE16011 dataset. (XLSX 22 kb)

## Abbreviations

ATRX: X - linked alpha thalassemia mental retardation syndrome gene
AUC: Area under the curve
BP: Biological Process
CC: Cellular Component
EGFR: Epidermal growth factor receptor
FPKM: Fragments per kilobase per million
GBM: Glioblastoma multiforme
GEO: Gene Expression Omnibus
GO: Gene Ontology
GS: Gene significance
IDH1: Isocitrate dehydrogenase 1
IDH2: Isocitrate dehydrogenase 2
IGF-1: Insulin-like growth factor 1
KEGG: Kyoto Encyclopedia of Genes and Genomes
MED10: Mediator complex subunit 10
MF: Molecular Function
MGMT: O6­methylguanine ­ DNA methyltransferase
MS: Module significance
OSMR: Oncostatin M receptor
PDGFRA: Platelet-derived growth factor receptor alpha
PTPRN: Protein tyrosine phosphatase, receptor type N
ROC: Receiver operating characteristic curve
SOX21: SRY (sex determining region Y)-box 21
TCGA: The Cancer Genome Atlas
TOM: Topological overlap measure
VEGF: Vascular endothelial growth factor precursor
WGCNA: Weighted Gene Co-expression Network Analysis
