# Supplementary material for: Identification of glioblastoma gene prognosis modules based on weighted gene co-expression network analysis
Source: BMC Med Genomics. 2018 Nov 1;11:96. doi: 10.1186/s12920-018-0407-1 (PMC6211550; doi:10.1186/s12920-018-0407-1)
Supplement: Supplementary file 6 — Table S1. Top ten prognostic genes identified from Cox regression analysis. (DOCX 21 kb) [file 12920_2018_407_MOESM6_ESM.docx]

| Gene | HR | 95%CI | Pvalue | Description | Function |
| --- | --- | --- | --- | --- | --- |
| PTPRN | 1.44 | 1.23-1.69 | 6.98E-06 | A member of the protein tyrosine phosphatase family | Response to reactive oxygen species; [Response to insulin](http://amigo.geneontology.org/amigo/term/GO:0032868); |
| MED10 | 3.53 | 1.92-6.5 | 5.18E-05 | A component of the Mediator complex | Transcription initiation from RNA polymerase II promoter |
| OSMR | 1.44 | 1.2-1.75 | 0.00012604 | A member of the type I cytokine receptor family | Regulation of cell proliferation;  Regulation of acute inflammatory response |
| EFEMP2 | 1.51 | 1.2-1.92 | 0.00050940 | Extracellular matrix proteins | Extracellular matrix organization |
| MXRA8 | 1.48 | 1.19-1.85 | 0.00053635 | Matrix-remodelling associated 8 | Establishment of glial blood-brain barrier |
| SOX21 | 0.69 | 0.56-0.86 | 0.00059626 | SRY(sex determining region Y)-box 21 | Regulation of transcription, DNA-templated; Stem cell differentiation |
| PDIA4 | 1.70 | 1.26-2.3 | 0.00059921 | Protein disulfide isomerase family A, member 4 | Protein secretion |
| PLAUR | 1.42 | 1.16-1.76 | 0.00096055 | The receptor for urokinase plasminogen activator | Regulation of protein phosphorylation; Urokinase plasminogen activator signaling pathway |
| UPP1 | 1.45 | 1.16-1.82 | 0.00098554 | Uridine phosphorylase 1 | Nucleobase-containing compound metabolic process |
| PTRF | 1.37 | 1.14-1.66 | 0.001013 | Polymerase I and transcript release factor | Termination of RNA polymerase I transcription; [Transcription initiation from RNA polymerase I promoter](http://amigo.geneontology.org/amigo/term/GO:0006361) |

Additional file 6: **Table S1** Top ten prognostic genes identiﬁed from Cox regression analysis
